# Exploration of hub genes involved in PCOS using biological informatics methods

**DOI:** 10.1097/MD.0000000000030905

**Published:** 2022-10-07

**Authors:** Fei Zhou, Yuling Xing, Tiantian Cheng, Linlin Yang, Huijuan Ma

**Affiliations:** a Department of Internal Medicine, Hebei Medical University, Shijiazhuang, Hebei, China; b Key Laboratory of Metabolic Diseases, Hebei General Hospital, Shijiazhuang, Hebei, China; c Department of Endocrinology, Hebei General Hospital, Shijiazhuang, Hebei, China.

**Keywords:** bioinformatics analysis, differentially expressed genes, PI3K/Akt pathway, polycystic ovarian syndrome

## Abstract

The aim of this study was to find underlying genes and their interaction mechanism crucial to the polycystic ovarian syndrome (PCOS) by analyzing differentially expressed genes (DEGs) between PCOS and non-PCOS subjects.

**Methods:**

Gene expression data of PCOS and non-PCOS subjects were collected from gene expression omnibus (GEO) database. GEO2R were used to calculating *P* value and logFC. The screening threshold of DEGs was *P* < .05 and | FC | ≥ 1.2. GO annotation and Kyoto encyclopedia of genes and genomes (KEGG) signaling pathway enrichment analysis was performed by using DAVID (2021 Update). The protein-protein interaction (PPI) network of DEGs was constructed by using the STRING database, and the hub genes were recognized through Hubba plugin of Cytoscape software.

**Results:**

PCOS and non-PCOS subjects shared a total of 174 DGEs, including 14 upregulated and 160 downregulated genes. The GO biological processes enriched by DEGs mainly involved actin cytoskeleton organization, positive regulation of NF-κB signaling pathway, and positive regulation of canonical Wnt signaling pathway. The DEGs were significantly enriched in cytoplasm, nucleus and cytosol. Their molecular functions mainly focused on protein binding, calmodulin binding and glycerol-3-phosphate dehydrogenase activity. The PI3K/Akt signaling pathway and glycosaminoglycan biosynthesis were highlighted as critical pathways enriched by DEGs. 10 hub genes were screened from the constructed PPI network, of which EGF, FN1 and TLR4 were mainly enriched in the PI3K/Akt signaling pathway.

**Conclusion:**

In this study, a total of 174 DEGs and 10 hub genes were identified as new candidate targets for insulin resistance (IR) in PCOS individuals, which may provide a new direction for developing novel treatment strategies for PCOS.

## 1. Introduction

Polycystic ovary syndrome (PCOS), one of the most common endocrine disorders, is affecting up to 5% to 10% of women worldwide.^[1]^ PCOS is an enigmatic endocrine disorder caused by hormonal imbalances and usually presents reproductive, metabolic and psychological syndromes.^[2]^ According to the Rotterdam ESHRE/ASRM Consensus, the diagnostic criteria of PCOS are hyperandrogenism, ovulation disorder, polycystic ovaries (detected by ultrasonography) and the exclusion of other endocrinopathies.^[3]^ The current understanding of the multisystemic features of this syndrome is increasing.^[4,5]^ PCOS not only increases the risk of pregnancy-related disorders such as miscarriage, gestational diabetes, preterm birth and preeclampsia,^[6–8]^ but also causes a range of other health problems, including chronic inflammation, insulin resistance (IR), glucose intolerance, metabolic syndrome and hypertension.^[9]^ Patients with PCOS often exhibit type 2 diabetes mellitus (T2DM), obesity and other metabolic disorders.^[10]^ PCOS is no longer simply considered an ovarian disease.^[11]^ Skeletal muscle IR has been reported in >60% of patients with PCOS and 10% of women with PCOS may develop T2DM by the age of 40 years.^[12]^ However, the molecule mechanism of IR in PCOS remains unclear and is worthy of further study. In this study, microarray data of PCOS and non-PCOS subjects were analyzed by using bioinformatics methods, which helped to explore critical differentially expressed genes (DEGs) underlying the pathogenesis of IR in PCOS.

We present the following article in accordance with the MDAR reporting checklist.

## 2. Materials and methods

### 2.1. Sources of microarray data

Microarray data of PCOS and non-PCOS subjects were obtained from the gene expression omnibus (GEO, https://www.ncbi.nlm.nih.gov/geo/) database by setting keywords as: “Syndrome, Polycystic Ovary” OR “Stein-Leventhal Syndrome” OR “Stein Leventhal Syndrome” OR “Syndrome, Stein-Leventhal” OR “Sclerocystic Ovarian Degeneration” OR “Ovarian Degeneration, Sclerocystic” OR “Sclerocystic Ovary Syndrome” OR “polycystic ovarian syndrome” OR “Ovarian Syndrome, Polycystic” OR “Polycystic Ovary Syndrome 1” OR “Sclerocystic Ovaries” OR “Ovary, Sclerocystic” OR “Sclerocystic Ovary” OR “PCOS” OR “polycystic ovarian syndrome”. GSE6798 (including 13 muscle samples from healthy control subjects and 16 muscle samples from PCOS patients) and GSE8157 (including 13 muscle samples from healthy control subjects and 10 muscle samples from PCOS patients) were downloaded for further analysis. Both datasets were based on GPL570 [HG-U133_PLus_2] Affymetrix Human Genome U133 Plus 2.0 Array chip. In addition, the PCOS patients meet the elevated free testosterone levels (>0.035 nmol/L) and hyperinsulinemia (>85 pmol/L); and the CON subjects meet regular menses, normal glucose tolerance, and no family history of diabetes. The study was conducted in accordance with the Declaration of Helsinki (as revised in 2013).

### 2.2. Data processing and DEGs screening

GEO2R was used to calculate the logFC (log2foldchange) and *P* value of each gene. DEGs were identified according to the cutoff of *P* < .05 and |FC| ≥1.2 (|logFC|≥0.3). The DEGs with *P* < .05 and FC ≥ 1.2 (logFC ≥ 0.3) were considered to be up-regulated genes, while the DEGs with *P* < .05 and FC ≤ 1.2 (logFC ≤ 0.3) were considered to be down-regulated genes.

Common upregulated and downregulated genes between the two datasets were identified and defined as Co-differentially expressed genes (Co-DEGs). The Co-DEGs in the two datasets were displayed by Venn, and the volcano plots and heatmaps were drawn by using R-related visualization capabilities.

### 2.3. Functional enrichment analysis of DEGs

Database for annotation, visualization, and integrated discovery (DAVID) (2021 Update) was used for gene ontology (GO) annotation and Kyoto encyclopedia of genes and genomes (KEGG) pathway enrichment analysis. GO annotation is composed of biological processes (BP), cellular components (CC) and molecular functions (MF), and it is a good tool which can help to predict the main protein functions of DEGs. KEGG signaling pathway enrichment analysis is capable to classify all kinds of DEGs to specific pathways with system path. The DEGs were uploaded to DAVID (2021 Update) for GO annotation and KEGG signaling pathway analysis, then the top 10 GO annotation results were selected for bubble chart plotting.

### 2.4. Construction of protein-protein interaction (PPI) network between DEGs and HUB gene identification

The PPI network of DEGs was constructed with the criterion of “confidence >0.7” based on the STRING (Search Tool for the Retrieval of Interacting Genes/Proteins, https://string-db.org/) database. The algorithm of the Hubba plugin of Cytoscape v3.9.1 was used for the top 10 hub genes analysis in the constructed PPI network.

## 3. Results

### 3.1. Differentially expressed genes (DEGs)

**Figure 1.**
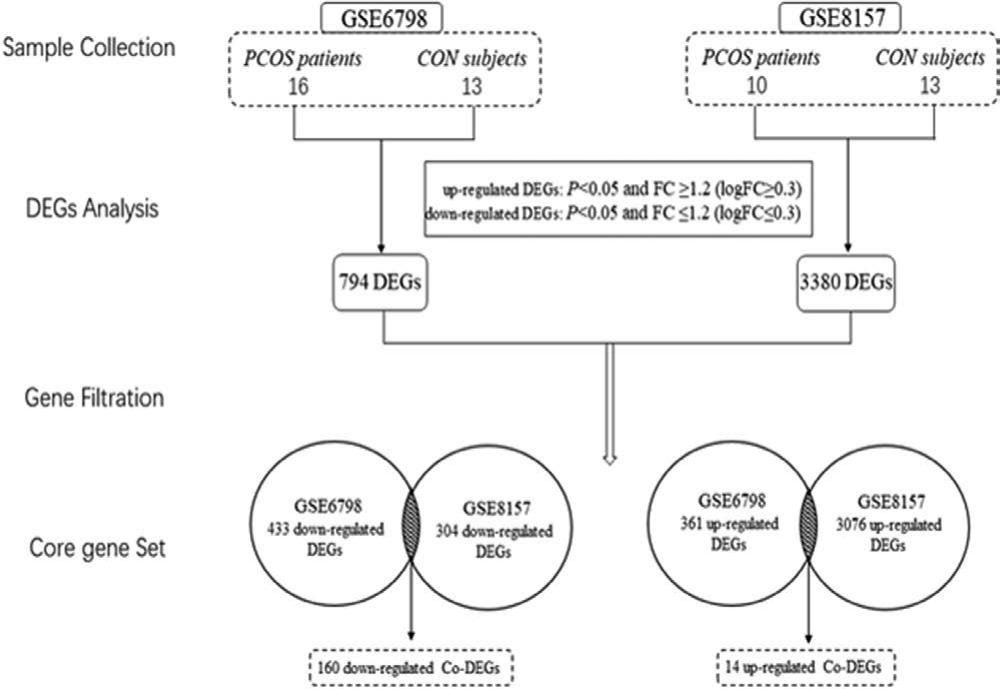
The flow diagram of the process of gene differential analysis.

**Figure 2.**
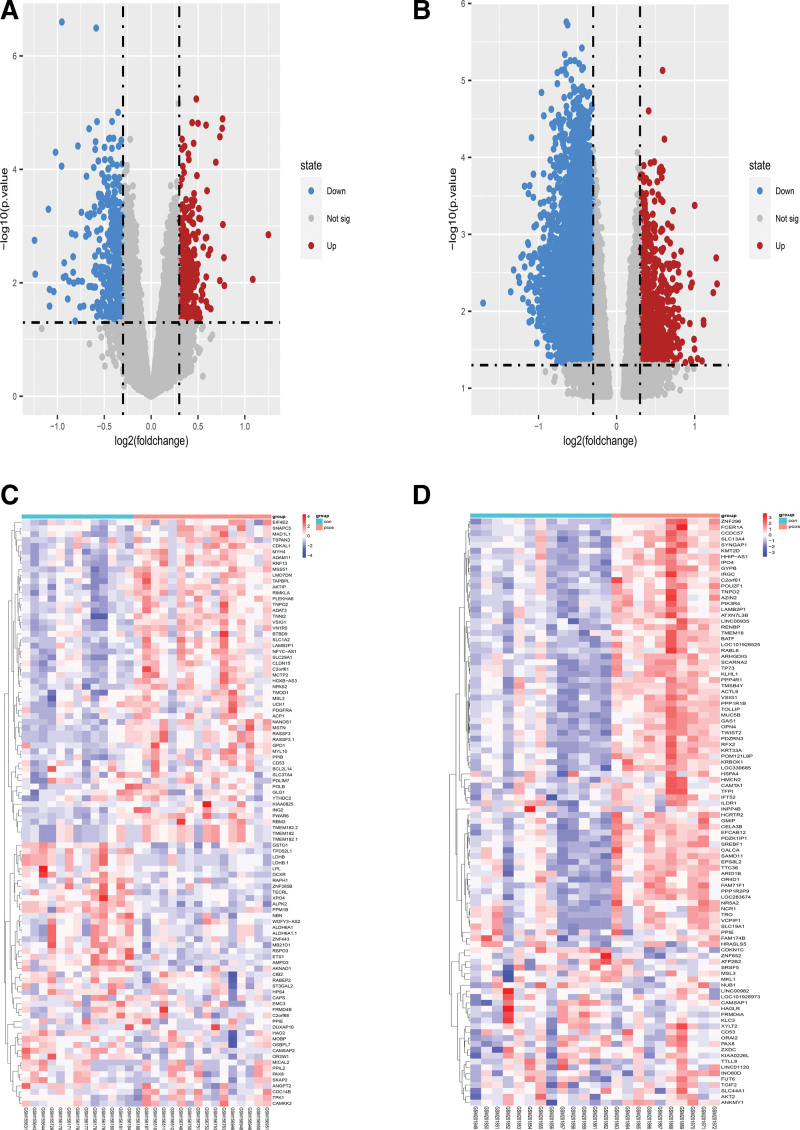
Volcano plots and heatmaps of DEGs in GSE6798 and GSE8157. The volcano plots showed the DEGs in the chip compared with normal umbilical cords; red dots represent PCOS highly expressed genes, and blue dots represent PCOS low expressed genes. The heatmaps showed the top 100 genes with the most significant DEGs. Red represents a high expression signal, and blue represents a low expression signal. (A) GSE6798 differential expression gene volcano plot. (B) GSE8157 differential expression gene volcano plot. (C) GSE6798 differential expression gene heatmap. (D) GSE8157 differential expression gene heatmap. DEGs = differentially expressed genes, PCOS = polycystic ovarian syndrome.

GSE6798 contained 794 differentially expressed genes, including 433 down-regulated genes and 361 up-regulated genes. GSE8157 contained 3380 differentially expressed genes, including 304 down-regulated genes and 3076 up-regulated genes. GSE6798 and GSE8157 shared 174 Co-DEGs, including 14 up-regulated genes and 160 down-regulated genes (Table 1, Fig. 1). Volcano plots and heatmaps of DEGs were shown in Figure 2.

**Table 1:** Co-differential genes between PCOS patients and CON subjects.

PCOS vs CON	Gene symbol
Up regulated genes (14)	DOCK2,ZSCAN30,DMKN,RERG,THADA,ST6GAL2,RASEF,SLAMF7,CC2D2B,MIR4296,HAS2,PPP1R16A,ERVH-4,IFT57
Down regulated genes (160)	GAPDHS,ATP9B,C12orf66,EIF4G3,MIR6787///SLC16A3,MIB2,CCDC57,TNNT3,IPO4,ATXN7L3B,PDZRN3,GPD1,ZZEF1,DDX49,CD53,NBPF3,LONRF2,AHCYL1, IRX3,KCTD7///RABGEF1,NFYC,CCDC6,NDRG2,EHBP1L1,SGF29,EGF,VSIG1,SYCE3,C2orf61,CYB561,CBWD7///CBWD6///CBWD3///CBWD5///CBWD2///CBWD1,MARVELD3,FN1,EIF5A,TGIF2,TRIM42,MCTP2,PKP2,CA12,CRTAP,HES2,DUSP3,AMFR,MYEOV,EEF1D,OSBPL7,PIF1,TGFBRAP1,HBG2///HBG1,ANKRD44,ARHGEF34P///OR2A7///OR2A4,MOBP,SMIM11A,ZBTB10,B3GALT6,NMRK2,SLC29A1,SH2D1B,KRBOX1,TFPI,SMTN,GAS1,GOSR2,EPS8L2,RAB40C,C2orf68,ACP1,GPC3,PDGFRA,ARHGAP36,BCRP3,LOC100653137///CDH23,MYH4,SOBP,FCGR3B///FCGR3A,HCRT,TNPO2,NEMP1,MAFF,ACY3,ZNF652,SLC22A18AS,DLGAP1AS1,RASSF3,TPSG1,VCPIP1,TSPAN3,MSL3,CIB2,SNURF///SNRPN,CFAP46,RAPH1,REST,ATP11AAS1,LAMB2P1,FAM224A///FAM224B,UHMK1,PPP4R3B,ADGRL1,RAPGEF1,FRMD6,LMO2,LZTS1,HS6ST2,LINC01091,MAD1L1,LOC100996843///KCNJ18///KCNJ12,OPRL1,CCNYL1,KLC3,PRKAG3,TUBB3,ASMTL,AS1,B3GLCT,POLB,FAM120C,PRKCE,MOV10,VN1R5,STARD6,PIAS2,CAPZA1PSAP,INPP4B,HIBCH,SURF1,EFHB,TPGS2,USP21,ENSA,RHOB,FAIM2,FAR1,ADAT3,PAX8,TRIM71,WRN,HIST1H2BC///HIST1H2BI///HIST1H2BE///HIST1H2BF///HIST1H2BG,TLR4,MS4A7,CYP2A7P1,GPD2,FRZB,CAMKK2,LINC01010,PPIE,COL6A1,FAM224A,SV2C,MKL1,NANOS1,ESPN,PGD,PTPN13,PPP4R4,HIPK1,TTC7B,LMBR1,SLC16A10,ANKRD23

PCOS = polycystic ovarian syndrome.

### 3.2. Functional analysis of DEGs

**Figure 3.**
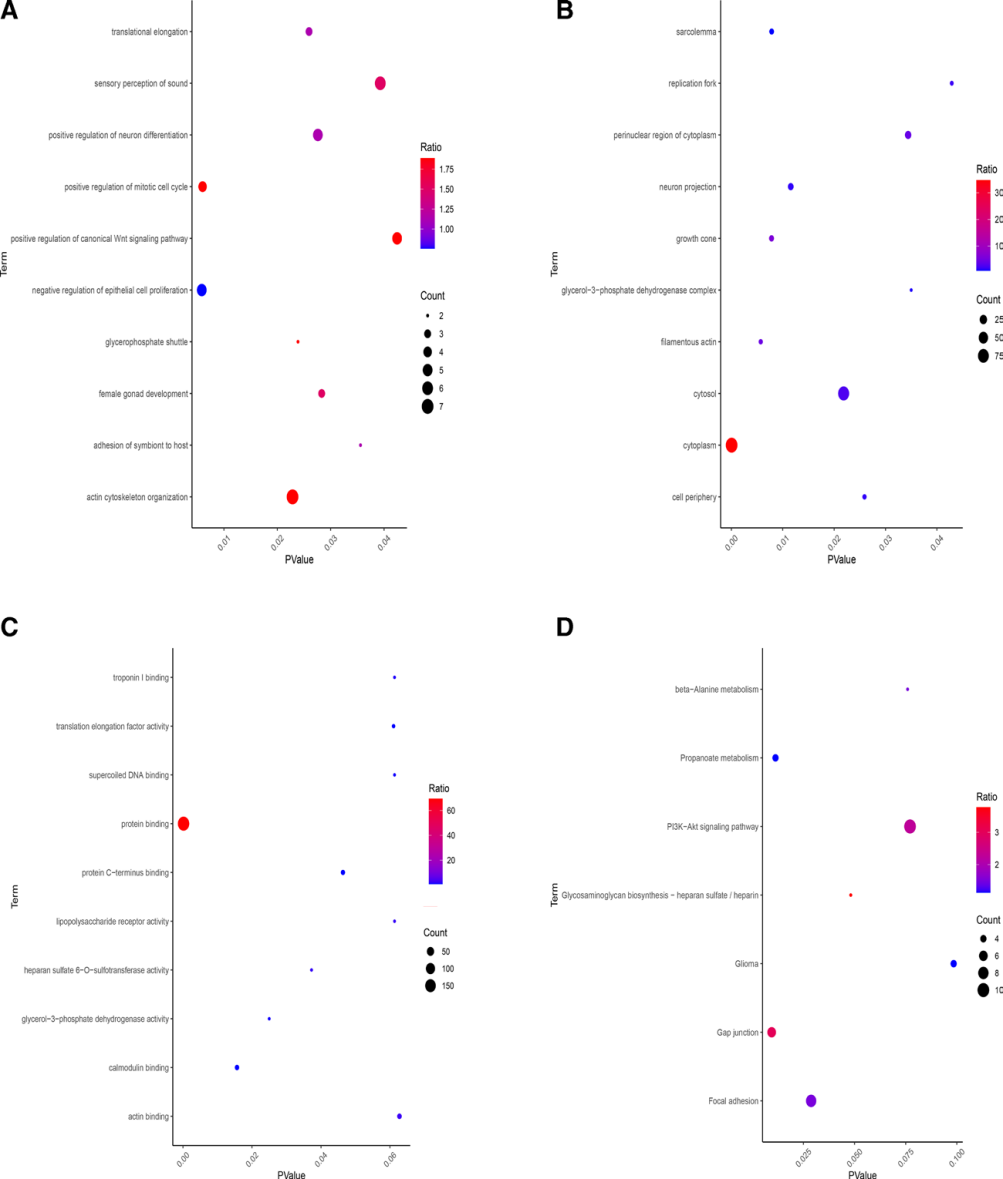
GO enrichment analysis and KEGG pathway enrichment results of DEGs. The abscissa represents the *P* value, and the ordinate represents different terms. The larger the dots in the figure, the more genes contained in this term; the redder the dot color, the higher the probability of genes rich in this term. (A) Top 10 enrichment analysis results of BP. (B) Top 10 enrichment analysis results of CC. (C) Top 10 enrichment analysis results of MF. (D) KEGG pathway enrichment results of DEGs. BP = biological processes, CC = cellular component, DEGs = differentially expressed genes, KEGG = Kyoto encyclopedia of genes and genomes, MF = molecular function.

GO annotation and KEGG signaling pathway enrichment analysis were performed on the screened DEGs using DAVID (2021 Update) online tool. The GO annotation includes BP, CC, and MF, and the corresponding bubble chart was shown in Figure 3. The Top 10 BP enriched by DEGs mainly included actin cytoskeleton organization, sensory perception of sound, positive regulation of I-kappaB kinase/NF-kappaB signaling, negative regulation of epithelial cell proliferation, positive regulation of neuron differentiation, positive regulation of canonical Wnt signaling pathway, small GTPase mediated signal transduction, neuron projection development, peptidyl-tyrosine phosphorylation and positive regulation of mitotic cell cycle. CC results showed that these genes were mainly involved in cytoplasm, nucleus, cytosol, nucleoplasm, perinuclear region of cytoplasm, centrosome, neuron projection, growth cone, sarcolemma and Z disc. Their MF mainly focus on protein binding, actin binding, calmodulin binding, protein C-terminus binding, guanyl-nucleotide exchange factor activity, transcription coactivator activity, GDP binding, translation elongation factor activity, ribonucleoprotein complex binding and protein phosphatase regulator activity (Table 2).

**Table 2:** GO enrichment analysis of DEGs.

Category	Term	Description	Count	*P* value
GOTERM_BP_DIRECT	GO:0030036	Actin cytoskeleton organization	7	.022849453
GOTERM_BP_DIRECT	GO:0007605	Sensory perception of sound	6	.039294051
GOTERM_BP_DIRECT	GO:0043123	Positive regulation of I-kappaB kinase/NF-kappaB signaling	6	.080721309
GOTERM_BP_DIRECT	GO:0050680	Negative regulation of epithelial cell proliferation	5	.005836237
GOTERM_BP_DIRECT	GO:0045666	Positive regulation of neuron differentiation	5	.027598005
GOTERM_BP_DIRECT	GO:0090263	Positive regulation of canonical Wnt signaling pathway	5	.042450194
GOTERM_BP_DIRECT	GO:0007264	Small GTPase mediated signal transduction	5	.059577758
GOTERM_BP_DIRECT	GO:0031175	Neuron projection development	5	.061027091
GOTERM_BP_DIRECT	GO:0018108	Peptidyl-tyrosine phosphorylation	5	.07489969
GOTERM_BP_DIRECT	GO:0045931	Positive regulation of mitotic cell cycle	4	.005992108
GOTERM_CC_DIRECT	GO:0005737	Cytoplasm	92	5.76951E-05
GOTERM_CC_DIRECT	GO:0005634	Nucleus	81	.067649363
GOTERM_CC_DIRECT	GO:0005829	Cytosol	79	.02185023
GOTERM_CC_DIRECT	GO:0005654	Nucleoplasm	57	.050518681
GOTERM_CC_DIRECT	GO:0048471	Perinuclear region of cytoplasm	16	.034396399
GOTERM_CC_DIRECT	GO:0005813	Centrosome	12	.057334082
GOTERM_CC_DIRECT	GO:0043005	Neuron projection	11	.011558785
GOTERM_CC_DIRECT	GO:0030426	Growth cone	7	.007824459
GOTERM_CC_DIRECT	GO:0042383	Sarcolemma	6	.007846244
GOTERM_CC_DIRECT	GO:0030018	Z disc	5	.071376467
GOTERM_MF_DIRECT	GO:0005515	Protein binding	184	.000163413
GOTERM_MF_DIRECT	GO:0003779	Actin binding	9	.062744136
GOTERM_MF_DIRECT	GO:0005516	Calmodulin binding	8	.015655052
GOTERM_MF_DIRECT	GO:0008022	Protein C-terminus binding	7	.046368853
GOTERM_MF_DIRECT	GO:0005085	Guanyl-nucleotide exchange factor activity	7	.072332123
GOTERM_MF_DIRECT	GO:0003713	Transcription coactivator activity	7	.0994205
GOTERM_MF_DIRECT	GO:0019003	GDP binding	4	.073033471
GOTERM_MF_DIRECT	GO:0003746	Translation elongation factor activity	3	.061042836
GOTERM_MF_DIRECT	GO:0043021	Ribonucleoprotein complex binding	3	.071473186
GOTERM_MF_DIRECT	GO:0019888	Protein phosphatase regulator activity	3	.078723052

**Figure 4.**
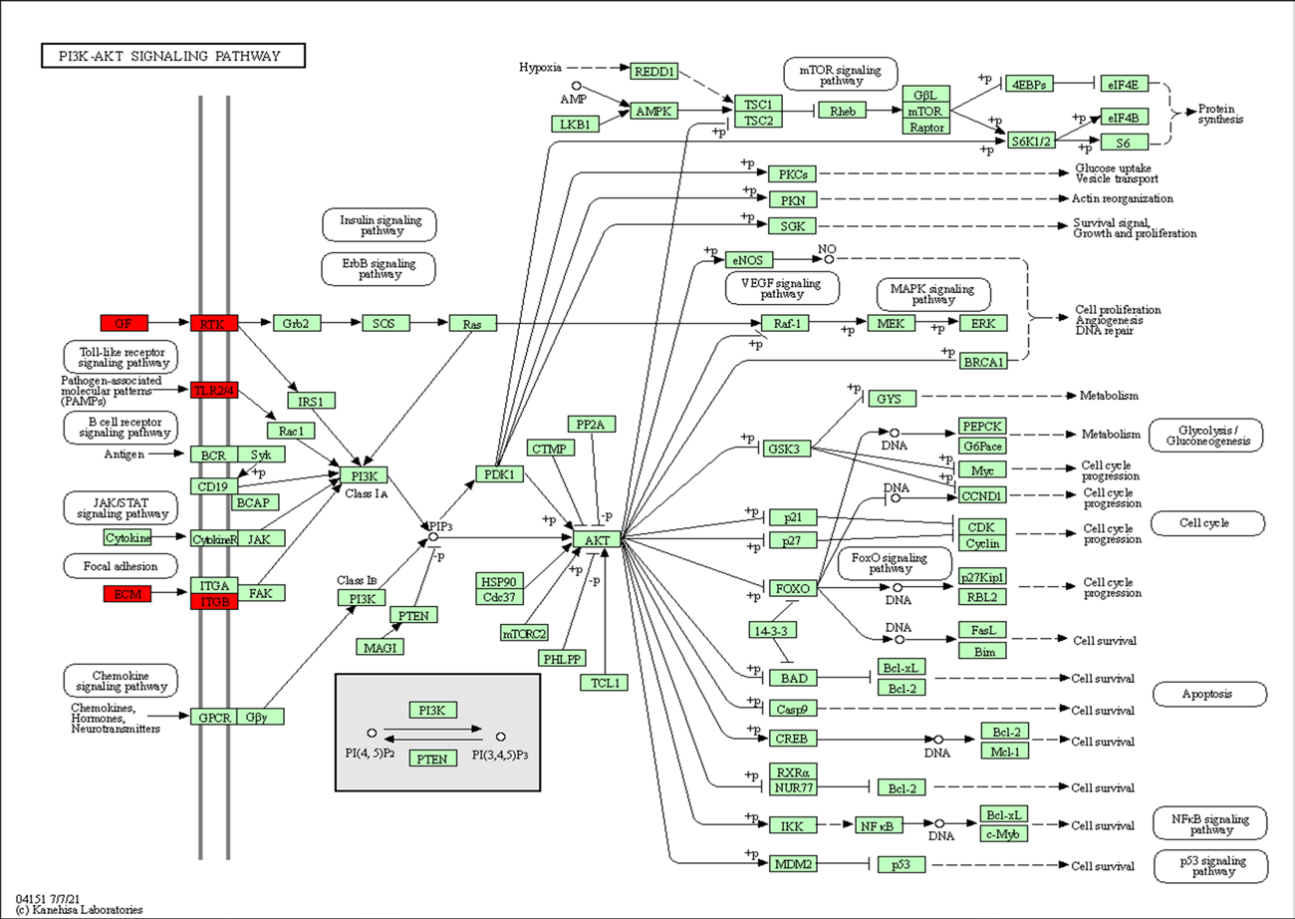
PI3K/Akt signaling pathway. Red represents the hub genes we identified in the PI3K/Akt signaling pathway.

In total, 7 KEGG signaling pathways were enriched by DEGs, including PI3K-Akt signaling pathway, Focal adhesion, Gap junction, Propanoate metabolism, Glioma, Glycosaminoglycan biosynthesis and beta-Alanine metabolism. Corresponding detailed data were shown in Figure 3 D and Table 3. Interestingly, PI3K/Akt pathway is commonly correlated with insulin resistance, a major pathogenic factor for IR (Fig. 4).

**Table 3:** KEGG pathway enrichment analysis of DEGs.

Category	Term	Description	Count
KEGG_PATHWAY	hsa04151	PI3K-Akt signaling pathway	10
KEGG_PATHWAY	hsa04510	Focal adhesion	8
KEGG_PATHWAY	hsa04540	Gap junction	6
KEGG_PATHWAY	hsa00640	Propanoate metabolism	4
KEGG_PATHWAY	hsa05214	Glioma	4
KEGG_PATHWAY	hsa00534	Glycosaminoglycan biosynthesis	3
KEGG_PATHWAY	hsa00410	beta-Alanine metabolism	3

KEGG = Kyoto encyclopedia of genes and genomes.

### 3.3. Core network of PCOS

**Figure 5.**
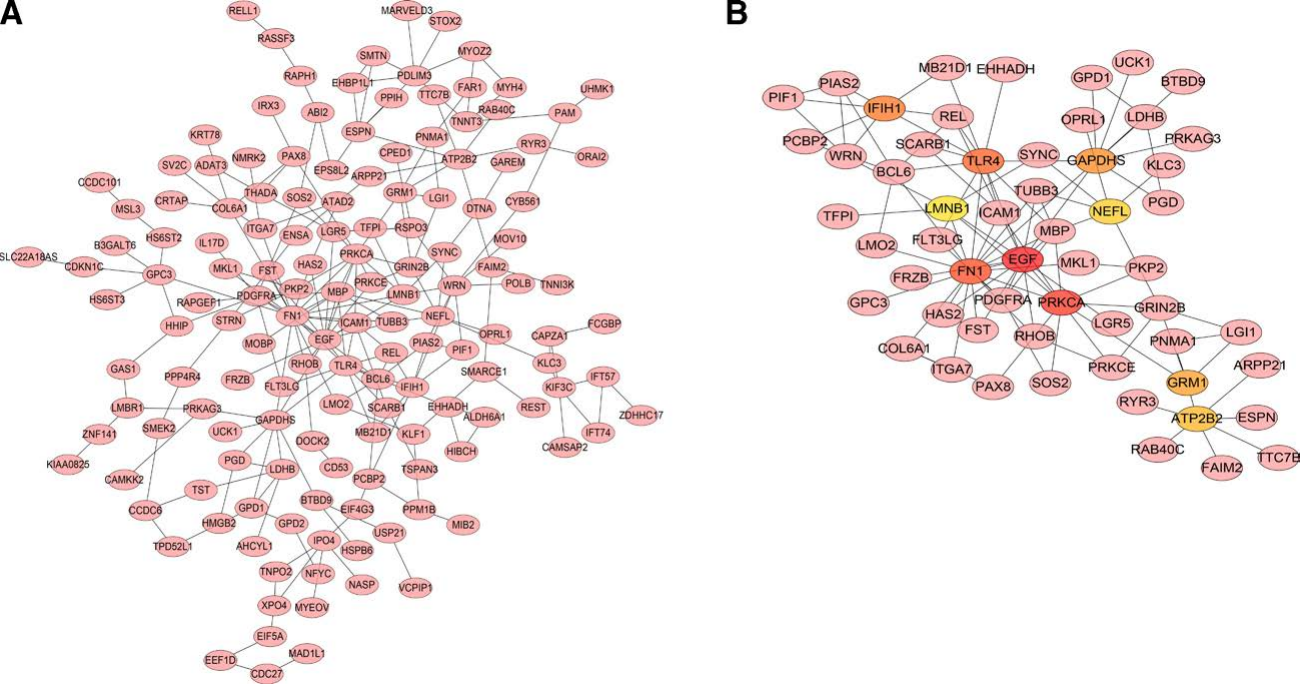
PPI network of DEGs and hub genes. (A) PPI network of DEGs. Pink nodes represent the interaction among DEGs. Only the 148 DEGs that interact with other ones were demonstrated in the network. (B)Top 10 hub genes identified from the PPI network. From the red nodes to the yellow ones, the connection degree of each molecule with others gradually decreases. DEGs = differentially expressed genes, PPI = protein-protein interaction.

Based on the DEGs between PCOS and non-PCOS subjects, the PPI network of PCOS was constructed using the STRING database, which was displayed in Figure 5A. The constructed network of PCOS is comprised of 148 nodes and 215 interaction edges. The degree of connectivity of each gene was calculated using the Hubba plugin of Cytoscape V3.9.1. Accordingly, Top10 genes with the highest degree were obtained, which included EGF, PRKCA, FN1, TLR4, IFIH1, GAPDHS, GRM1, ATP2B2, NEFL, LMNB1. To further construct the core network of PCOS, these hub genes and their neighbors were extracted and reconnected. Finally, we obtained a core network of PCOS comprised of 57 nodes and 103 edges (Fig. 5B).

## 4. Discussion

PCOS is defined as a highly complicated endocrine disease in women, whereas the etiology and physiopathology have not been elucidated sufficiently.^[13,14]^ Currently, IR in PCOS patients is a hot topic in the field of endocrinology and IR appears to be the fundamental key factor within the pathophysiology of PCOS.^[15]^ Numerous studies have confirmed that high fasting glucose and increased serum insulin are commonly found in PCOS patients.^[16,17]^ Impaired insulin responsiveness has been observed in skeletal muscle and myotubes of PCOS women.^[18]^ The skeletal muscle accounts for the largest portion of insulin-mediated whole-body glucose disposal,^[19]^ and thus, skeletal muscle IR is crucial for whole-body IR and PCOS. However, the research on the molecular level of IR in PCOS is lacking. In the present study, bioinformatics methods were used for analyzing the gene expression microarray data extracted from PCOS and non- PCOS skeletal muscle tissue. Ultimately, we detected a total of 174 co-DEGs by analyzing the gene expression microarray data from GSE6798 and GSE8157 for further study.

The GO analysis results revealed that the genes with significant differences in expression were mainly involved in positive regulation of NF-κB signaling pathway, positive regulation of canonical Wnt signaling pathway and protein binding. It has been pointed out that the activated NF-κB signaling pathway might be one of the important factors in the pathogenesis of IR in PCOS.^[20,21]^ Conversely, IR can also disrupt ovarian and uterine glucocorticoid receptor activation by regulating the NF-κB signaling pathway and then lead to PCOS.^[22]^ Zhao, Y et al demonstrated that the expression of WNT5a in PCOS patients was significantly elevated, and the up-regulated expression of WNT5a in PCOS increases inflammation and oxidative stress predominantly via the PI3K/Akt/NF-κB signaling pathway.^[23]^ In addition, previous studies have shown that the activation of WNT2/β-Catenin signaling pathway, was tightly associated with insulin resistance and estrogen deficiency, two hallmarks of PCOS.^[24]^ The Wnt signaling pathway is believed to be a significant contributor to the regulation of ovarian steroidogenesis, which could be one of the pathways modulated by gonadotropin signaling,^[25]^ and the cyclical changes of the endometrium are controlled by estrogen and progesterone via modulating the Wnt/β-catenin signaling pathway.^[26]^ Cytokine synthesis and increased endometrial inflammation in PCOS patients are coupled to the NFκB signaling pathway.^[27]^ The Wnt signal pathway might be involved in the apoptosis of granulosa cells and the progression of PCOS.^[28,29]^

Our KEGG enrichment analysis results suggested that many of EDGs were associated with the PI3K/Akt signaling pathway and glycosaminoglycan (GAG) biosynthesis, what is worth mentioning is that the PI3K/Akt signaling pathway was shown as the most enriched pathway. The PI3K/Akt signaling pathway is necessary for insulin stimulation of glucose transport,^[30,31]^ the impaired PI3K/Akt signaling pathway has been implicated in the development of IR.^[32]^ In turn, IR would exacerbate the PI3K/Akt signaling pathway, forming a vicious cycle.^[33]^ Thus, the PI3K/Akt signaling pathway may act as a molecular link between PCOS and IR.

The PPI network of the hub genes further revealed that EGF, FN1, and TLR4 all participate in the PI3K/Akt signaling pathway, and they were shown to be significantly decreased in the skeletal muscle of PCOS patients. This is consistent with previous animal studies. Evidence has shown that mRNA expression of EGF was reduced in PCOS rats.^[34,35]^ The decrease of EGF and consequent inhibition of its downstream PI3K/Akt signaling pathway might be a feasible mechanism for IR induced by PCOS. The hub gene fibronectin (FN1), an integral component of mammalian extracellular matrices (ECM), is an essential glycoprotein that has numerous biological functions.^[36]^ FN1 not only regulates adhesion, motility, growth and development^[37]^ but also is related to metabolic syndrome (MetS) and IR.^[38,39]^ In line with previous studies, the downregulation of FN1 seems to be related to PCOS^[40]^ and FN1 may regulate IR by affecting the PI3K/Akt signaling pathway.^[41]^ TLR4 is also a considerable hub gene, the downregulation of this gene can reduce the sensitivity of the insulin pathway by inhibiting the PI3K/AKT metabolic axis.^[42,43]^ Glycosaminoglycans are long unbranched and complex polysaccharides that are also an essential component of ECM. It is by now generally accepted that GAG plays an important role in cell adhesion, migration, survival, and apoptosis.^[44]^ Research has shown that GAG is increased in skeletal muscle of insulin-resistant mice,^[45]^ indicating it may play a key role in the pathophysiology of PCOS.

Although the genes we explored might be promising targets for PCOS which can help to provide new directions for the pathogenesis and its molecule mechanism of PCOS, it is also essential to conduct deeper and more extensive research for more accurate relevant clinical treatment.

## 5. Conclusion

In this study, a total of 174 DEGs and 10 hub genes were identified as new candidate targets for IR in PCOS individuals, and we indicated that the down-regulation of the PI3K/Akt signaling pathway may act as the underlying molecular basis of IR in PCOS, which may provide a new direction for developing novel treatment strategies for PCOS.

## Author contributions

Fei Zhou performed the data analysis and wrote this manuscript. Tiantian Cheng sorted out the data. Fei Zhou conceived and designed the experiments. Yuling Xing revised the manuscript. Linlin Yang and Huijuan Ma performed project coordination and supervised the project. All authors have seen and approved the final manuscript.

**Conceptualization:** Fei Zhou.

**Data curation:** Tiantian Cheng.

**Methodology:** Linlin Yang.

**Supervision:** Huijuan Ma.

**Writing – original draft:** Fei Zhou.

**Writing – review & editing:** Yuling Xing.
